# Gamma Knife Radiosurgery for Concurrent Trigeminal Neuralgia and Glossopharyngeal Neuralgia

**DOI:** 10.7759/cureus.20717

**Published:** 2021-12-26

**Authors:** Yoshiyasu Iwai, Kenichi Ishibashi, Kazuhiro Yamanaka

**Affiliations:** 1 Neurosurgery, Osaka City General Hospital, Osaka, JPN

**Keywords:** concurrent, radiosurgery, gamma knife, glossopharyngeal neuralgia, trigeminal neuralgia

## Abstract

An 82-year-old female had suffered right facial pain since 37 years of her age. The trigeminal neuralgia (TN) was controlled by carbamazepine and peripheral nerve block. The local block was effective for two to three years once performed, and as it became less effective, the patient took carbamazepine. Four months before gamma knife radiosurgery (GKRS), TN worsened. Analysis of her blood sample revealed autoimmune hemolytic anemia. It was suspected to be related to carbamazepine, and the patient stopped taking carbamazepine. The patient suffered pharyngeal pain and had difficulty swallowing for two months before GKRS. Tube feeding was started one month before GKRS. The patient was considered in pain due to TN and glossopharyngeal neuralgia (GPN). We performed GKRS continuously on the right cisternal portion of the trigeminal nerve at a maximum radiosurgical dose of 85 Gy for TN, and on the right cisternal portion of the glossopharyngeal nerve at a maximum dose of 80 Gy for GPN on the same day. The facial pain improved the day after GKRS. Seven days after treatment, the patient could swallow without pharyngeal pain, and the gastric tube was removed. Thirteen months after GKRS, the TN re-occurred but was controlled by carbamazepine 400 mg per day. GPN did not recur at that time. Simultaneous GKRS for concurrent TN and GPN is a less invasive and useful treatment option for non-candidates for surgical interventions.

## Introduction

Trigeminal neuralgia (TN) and glossopharyngeal neuralgia (GPN) are defined as neurovascular compression syndromes caused by vascular compression at the root exit zone of the cranial nerves [1]. Irritation of both the glossopharyngeal nerve and trigeminal nerve by arteries such as the superior cerebellar artery, anterior inferior cerebellar artery (AICA) and posterior inferior cerebellar artery (PICA) or the choroid plexus can result in concurrent TN and GPN [2-9]. Although cases of coexistence of TN and GPN have been previously reported [2-9], to the best of our knowledge, their simultaneous treatment by gamma knife radiosurgery (GKRS) has not been described. We experienced, and we report on, concurrent TN and GPN treated simultaneously by GKRS.

## Case presentation

**Figure 1:**
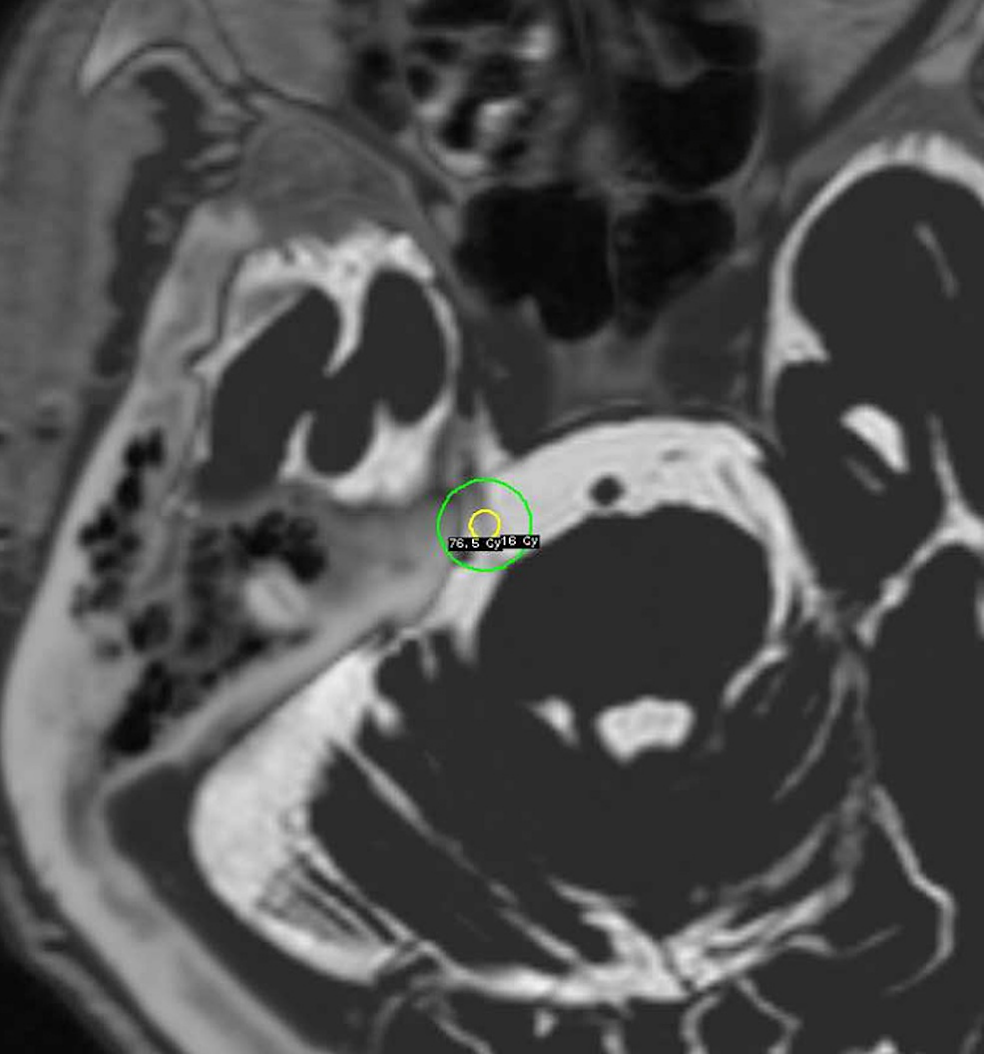
Dose plan of gamma knife radiosurgery by three-dimensional CISS images localized on MRI and CT under stereotactic conditions for trigeminal neuralgia CISS = Interference in steady state; MRI = Magnetic resonance imaging; CT = Computed tomography The cisternal portion of the trigeminal nerve, close to the Gasserian ganglion, was irradiated by a maximum dose of 85 Gy, using a single 4 mm collimator (yellow circle showing 90% isodose line and green circle showing 16 Gy isodose).

An 82-year-old female had suffered right facial pain since 37 years of her age. She experienced clusters of pain affecting the right cheek. The TN had been managed by a combination of medication by carbamazepine and peripheral nerve block. The local block was effective for two to three years once performed. When it became less effective, the patient took carbamazepine. Four months before GKRS, the patient suffered cholecystitis and was admitted to the hospital. From that time, TN worsened. Analysis of her blood sample revealed autoimmune hemolytic anemia. It was suspected to be related to carbamazepine, and the patient stopped taking carbamazepine. Two months before GKRS, the patient began experiencing mild recurrent paroxysmal pain as clusters of sharp stabs on the right side of the throat, which radiated to the right ear. The pain was triggered by eating, swallowing, talking, and laughing. Eating was particularly difficult. It became difficult for the patient to swallow, and tube feeding was started one month before GKRS. Also, the TN was not well controlled by nerve block. The patient was in pain due to GPN and TN and was referred to our department for GKRS treatment. The patient had been administered pregabalin 75 mg per day, baclofen 15 mg per day, and codeine phosphate 20 mg per day. Neurological examination was normal except for TN and GPN. Axial three-dimensional constructive interference in steady state (CISS) magnetic resonance (MR) images revealed that the transverse pontine vein was attached to the right cisternal portion of the trigeminal nerve, and the nerve was atrophied. The posterior inferior cerebellar artery was attached to the cisternal portion of the right glossopharyngeal nerve. We planned to perform GKRS for TN and GPN simultaneously. We performed GKRS on the cisternal portion of the right trigeminal nerve, close to the Gasserian ganglion, at a maximum dose of 85 Gy using a single 4 mm collimator because this portion of the trigeminal nerve was the most well-identified (Figure 1). The maximum irradiated dose of the brain stem was 15.2 Gy.

**Figure 2:**
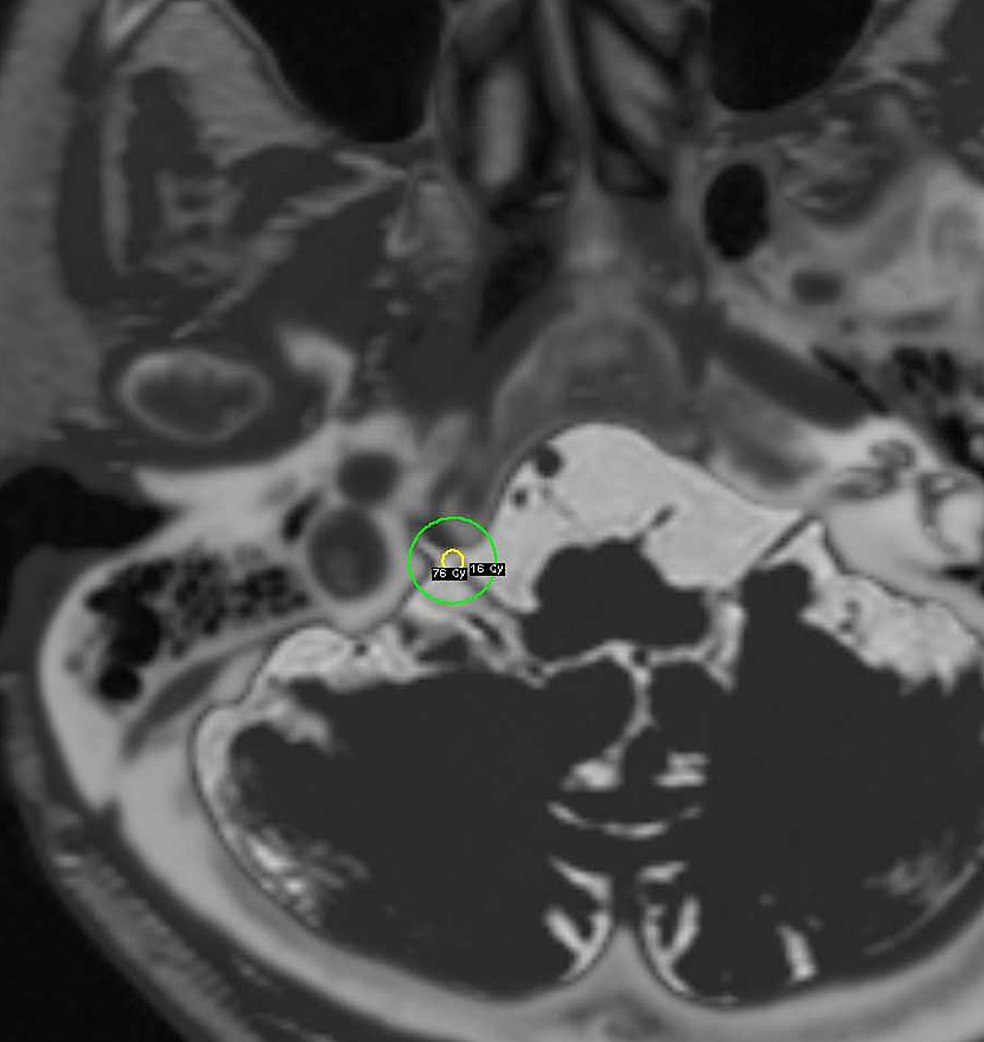
Dose plan of gamma knife radiosurgery by three-dimensional CISS images localized on MRI and CT under stereotactic conditions for glossopharyngeal neuralgia CISS = Interference in steady state; MRI = Magnetic resonance imaging; CT = Computed tomography The right cisternal portion of the glossopharyngeal nerve, close to the glossopharyngeal meatus of the jugular foramen, was irradiated by a maximum dose of 80 Gy, using a single 4 mm collimator (yellow circle showing 95% isodose line and green circle showing 16 Gy isodose)

We continuously performed GKRS on the right cisternal portion of the glossopharyngeal nerve, close to the glossopharyngeal meatus of the jugular foramen, at a maximum dose of 80 Gy localized on MRI and CT under stereotactic conditions on the same day (Figure 2). The maximum irradiated dose of the brain stem was 3.8 Gy. The facial pain improved the next day. Seven days after GKRS, the patient could swallow without pain, and the gastric tube was removed. Thirteen months after GKRS, TN re-occurred but was controlled by carbamazepine 400 mg per day. GPN did not recur at that time. Also, there were no side effects as facial sensory disturbance and swallowing disturbance at the follow-up periods.

## Discussion

We treated concurrent TN and GPN simultaneously by GKRS. The coincidence of TN and GPN was considered with the different etiologies of offending vessels or other pathology, the differing onsets of TN and GPN, and the treatment strategies. Concurrent GPN and TN in 15 patients was reported by Wang et al. including six patients with coexisting TN, GPN, and facial spasm (Table 1) [2-9].

**Table 1:** Reported cases of combined trigeminal and glossopharyngeal neuralgia TN = Trigeminal neuralgia; GPN = Glossopharyngeal neuralgia; SCA = Superior cerebellar artery; AICA = Anterior inferior cerebellar artery; PICA = Posterior inferior cerebellar artery; PV = Petrous vein; MVD = Microvascular decompression; op = Operation Laha et al. (1977) [2]; Yoshioka et al. (1985) [3]; Kobata et al. (1998) [4]; Warren et al. (2006) [5]; Katoh et al. (2012) [6]; Wang et al. (2014) [7]; Papalexopoulou et al. (2015) [8]; Maki et al. (2019) [9]

Authors/year	Age/Sex	Onset of symptoms	Cause of symptoms	Treatment	Outcome
Laha et al. (1977)	36/F	TN/GPN simultaneously	TN:SCA, GPN:PICA	MVD	Good
Yoshioka et al. (1985)	62/M	TN 35 years before GPN	TN:SCA, GPN: choroid plexus	TN:MVD, GPN: resection of choroid plexus	Complete remission	
Kobata et al. (1998)	67/M	GPN 11 months before TN	TN: SCA/AICA, GPN: PICA	MVD	Two patients: excellent	
	67/F	GPN 51 months before TN	TN: SCA/AICA, GPN:AICA/PICA	MVD	One patient; good	
	62/F	TN 92 months before GPN	TN:SCA/PV, GPN: PICA	MVD	-
Warren et al. (2006)	69/F	TN/GPN simultaneously	Lateral medullary infarction	Medication (clonazepan)	Significantly improved (three weeks)	
Katoh et al. (2012)	70/F	TN/GPN simultaneously	TN:SCA, GPN: PICA	MVD	Complete remission	
Wang et al. (2014)	61/M	TN/GPN simultaneously	TN:PV, GPN:PICA	MVD	Symptom disappeared immediately		
	56/M	TN/GPN simultaneously	TN:SCA, GPN: PICA	MVD	Symptom disappeared immediately		
	45/F	TN/GPN simultaneously	TN:SCA, GPN:PICA	MVD	TN relieved one week, GPN improved		
	54/F	TN two years before GPN (contralateral)	TN:SCA, GPN:PICA	MVD	Pain-free immediately	
	69/F	TN nine months before GPN(contralateral)	TN:SCA, GPN: PICA	MVD	TN relieved in two weeks, GPN improved		
	77/F	TN three months before GPN	TN: SCA, GPN: PICA	MVD	Symptoms disappeared immediately		
Papalexopolus et al. (2015)	78/M	GPN four years before TN	not mentioned	Glycerol rhizolysis of trigeminal ganglion	GPN recurrence after two years	
Maki et al. (2019)	68/M	TN op four years later GPN+TN	TN: Teflon ball, GPN: arachnoid adhesion	MVD	GPN disappeared, TN persisted		
Present case	82/F	TN 37 years before GPN	TN:PV, GPN: PICA	Gamma knife radiosurgery (GKRS)	GPN disappeared after seven days, TN improved after one day of GKRS, but recurred after one year		

TN was the first symptom in six patients, with onset intervals of three months to 35 years [3,4,7,9], and GPN was the first symptom in three patients, with onset intervals of 11 months to 51 months [4,8]. TN and GPN occurred simultaneously in six patients [2,5-7]. Vascular compression was identified in 12 patients. In one patient, GPN was caused by choroid plexus [3], in one patient arachnoid adhesion after microvascular decompression (MVD) for TN was speculated [9], and one patient was diagnosed with lateral medullary infarction [5]. MVD was performed on 12 patients, one patient was treated by glycerol rhizolysis of the trigeminal nerve [8], and one patient with medullary infarction was treated by clonazepam [5]. All the patients on whom MVD was performed reported pain relief after surgery. For the patient that received glycerol rhizolysis, GPN recurred two years after treatment and medical treatment was required [8].

The treatment options for TN and GPN include both medical and surgical therapy. General practice is to attempt to resolve patient symptoms by using drug therapy before resorting to surgery. If medical therapy fails to resolve the patient’s symptoms or if they become drug-intolerant, there are several surgical options. For TN, these include radiofrequency thermocoagulation, MVD [1], and GKRS [10,11]. Others are percutaneous balloon compression, glycerol rhizotomy, and extracranial peripheral denervation. For GPN, surgical treatments to be considered include microvascular decompression [2], percutaneous thermocoagulation, neurotomy by section of the glossopharyngeal nerve, and the upper rootlets of the vagus nerve, or GKRS [12-14]. Important factors to consider for each procedure are time until pain relief, length of response, recurrence rate, safety, side effects, availability, and cost, which vary for each procedure. We must also understand the effectiveness of GKRS for TN and GPN. GKRS is a minimally invasive neurosurgical approach. Its usefulness for classical trigeminal neuralgia has been established in long-term follow-up studies [10,11]. The effectiveness of GKRS for TN has been reported in literature where probabilities for maintaining pain relief were 46 to 64.9% for 5 years and 30 to 45.3% for 10 years [10,11]. Recently, the effectiveness of GKRS for GPN being the same as for TN has been recognized and reported [12-14]. Kano et al. reported adequate pain relief of 38% at three years, 38% at five years, and 28% at seven years, using a median maximum dose of 80 Gy [12]. Borius et al. reported using a median maximal dose of 85 Gy, where 17 (81%) patients were initially pain-free after GKRS. At three months, six months, and one year after GKRS, the percentages of patients with good outcomes were 87.6%, 100%, and 81.8%, respectively [14]. GKRS is a valuable, minimally invasive, surgical alternative for idiopathic GPN, with very high short-term and long-term efficacy, and without permanent complications as for TN. Especially in the case of GKRS for GPN, quality imaging, including T2 CISS/Fiesta MRI and bone CT acquisitions for good visualization of the nerve and the other bony anatomic landmarks, is essential for targeting accuracy and successful therapy [15]. A higher radiosurgical dose is effective for pain control but increases the risk of bother-some numbness in the case of TN. We use 85 Gy in GKRS for TN in our daily practice and we considered 85 Gy to be a reasonable dose of TN for this patient. As the optimal dose of GKRS for GPN was reported as 80 Gy [12] and 85 Gy [14], we used 80 Gy for GPN considering the effectiveness and complication. We understand the effectiveness of MVD and GKRS treatment strategies. The long-term effectiveness of MVD for TN is confirmed to be 70-75% at 10-year follow-ups [16,17]. The effectiveness of MVD for GPN is also confirmed to be around 85% [18]. We must understand that the effectiveness of GKRS for TN is inferior when compared to MVD, and that the effectiveness of GKRS for GPN is also inferior when compared to MVD. But our case was an elderly patient of 82 years, whose condition was unsuitable for surgical treatment due to autoimmune hemolytic anemia. We performed the less invasive treatment strategy of GKRS for TN and GPN simultaneously, and successfully controlled GPN and TN without complications.

## Conclusions

The coexistence of TN and GPN is extremely rare. The simultaneous GKRS treatment reported here for concurrent TN and GPN for the cisternal portion of the nerves using prescribed dose of 85 Gy for TN and a prescribed dose of 80 Gy for GPN is a less invasive and useful treatment option for non-candidates for surgical interventions.

## References

[1] Jannetta PJ (1980). Neurovascular compression in cranial nerve and systemic disease. Ann Surg.

[2] Laha RK, Jannetta PJ (1977). Glossopharyngeal neuralgia. J Neurosurg.

[3] Yoshioka J, Ueta K, Ohmoto T, Fujiwara T, Tabuchi K (1985). Combined trigeminal and glossopharyngeal neuralgia. Surg Neurol.

[4] Kobata H, Kondo A, Iwasaki K, Nishioka T (1998). Combined hyperactive dysfunction syndrome of the cranial nerves: trigeminal neuralgia, hemifacial spasm, and glossopharyngeal neuralgia: 11-year experience and review. Neurosurgery.

[5] Warren HG, Kotsenas AL, Czervionke LF (2006). Trigeminal and concurrent glossopharyngeal neuralgia secondary to lateral medullary infarction. AJNR Am J Neuroradiol.

[6] Katoh M, Aida T, Moriwaki T (2012). A case of combined glossopharyngeal and trigeminal neuralgia (Article in Japanese). No Shinkei Geka.

[7] Wang YN, Zhong J, Zhu J, Dou NN, Xia L, Visocchi M, Li ST (2014). Microvascular decompression in patients with coexistent trigeminal neuralgia, hemifacial spasm and glossopharyngeal neuralgia. Acta Neurochir (Wien).

[8] Papalexopoulou N, Hasegawa H, Selway R, Chong S, Ashkan K (2015). The treatment of combined trigeminal and glossopharyngeal neuralgia by glycerol rhizolysis of the trigeminal ganglion. Br J Neurosurg.

[9] Maki Y, Kikuchi T, Komatsu K, Takagi Y, Miyamoto S (2019). Rare case of concurrent glossopharyngeal and trigeminal neuralgia, in which glossopharyngeal neuralgia was possibly induced by postoperative changes following microvascular decompression for trigeminal neuralgia. World Neurosurg.

[10] Kondziolka D, Zorro O, Lobato-Polo J, Kano H, Flannery TJ, Flickinger JC, Lunsford LD (2010). Gamma knife stereotactic radiosurgery for idiopathic trigeminal neuralgia. J Neurosurg.

[11] Régis J, Tuleasca C, Resseguier N, Carron R, Donnet A, Gaudart J, Levivier M (2016). Long-term safety and efficacy of Gamma Knife surgery in classical trigeminal neuralgia: a 497-patient historical cohort study. J Neurosurg.

[12] Kano H, Urgosik D, Liscak R (2016). Stereotactic radiosurgery for idiopathic glossopharyngeal neuralgia: an international multicenter study. J Neurosurg.

[13] Pommier B, Touzet G, Lucas C, Vermandel M, Blond S, Reyns N (2018). Glossopharyngeal neuralgia treated by gamma knife radiosurgery: safety and efficacy through long-term follow-up. J Neurosurg.

[14] Borius PY, Tuleasca C, Muraciole X (2018). Gamma knife radiosurgery for glossopharyngeal neuralgia: a study of 21 patients with long-term follow-up. Cephalalgia.

[15] Moon WJ, Roh HG, Chung EC (2009). Detailed MR imaging anatomy of the cisternal segments of the glossopharyngeal, vagus, and spinal accessory nerves in the posterior fossa: the use of 3D balanced fast-field echo MR imaging. AJNR Am J Neuroradiol.

[16] Barker FG 2nd, Jannetta PJ, Bissonette DJ, Larkins MV, Jho HD (1996). The long-term outcome of microvascular decompression for trigeminal neuralgia. N Engl J Med.

[17] Sindou M, Leston J, Decullier E, Chapuis F (2007). Microvascular decompression for primary trigeminal neuralgia: long-term effectiveness and prognostic factors in a series of 362 consecutive patients with clear-cut neurovascular conflicts who underwent pure decompression. J Neurosurg.

[18] Rey-Dios R, Cohen-Gadol AA (2013). Current neurosurgical management of glossopharyngeal neuralgia and technical nuances for microvascular decompression surgery. Neurosurg Focus.

